# Advances in the development paradigm of biosample‐based biosensors for early ultrasensitive detection of alzheimer’s disease

**DOI:** 10.1186/s12951-021-00814-7

**Published:** 2021-03-09

**Authors:** Hem Prakash Karki, Yeongseok Jang, Jinmu Jung, Jonghyun Oh

**Affiliations:** 1 Department of Mechanical Design Engineering, College of Engineering, Jeonbuk National University, Jeonju, 54896 South Korea; 2 Department of Nano-bio Mechanical System Engineering, College of Engineering, Jeonbuk National University, Jeonju, 54896 South Korea

**Keywords:** Alzheimer’s disease, Biosample, Biomarker, Biosensor, Early detection

## Abstract

This review highlights current developments, challenges, and future directions for the use of invasive and noninvasive biosample-based small biosensors for early diagnosis of Alzheimer’s disease (AD) with biomarkers to incite a conceptual idea from a broad number of readers in this field. We provide the most promising concept about biosensors on the basis of detection scale (from femto to micro) using invasive and noninvasive biosamples such as cerebrospinal fluid (CSF), blood, urine, sweat, and tear. It also summarizes sensor types and detailed analyzing techniques for ultrasensitive detection of multiple target biomarkers (i.e., amyloid beta (Aβ) peptide, tau protein, Acetylcholine (Ach), microRNA137, etc.) of AD in terms of detection ranges and limit of detections (LODs). As the most significant disadvantage of CSF and blood-based detection of AD is associated with the invasiveness of sample collection which limits future strategy with home-based early screening of AD, we extensively reviewed the future trend of new noninvasive detection techniques (such as optical screening and bio-imaging process). To overcome the limitation of non-invasive biosamples with low concentrations of AD biomarkers, current efforts to enhance the sensitivity of biosensors and discover new types of biomarkers using non-invasive body fluids are presented. We also introduced future trends facing an infection point in early diagnosis of AD with simultaneous emergence of addressable innovative technologies.
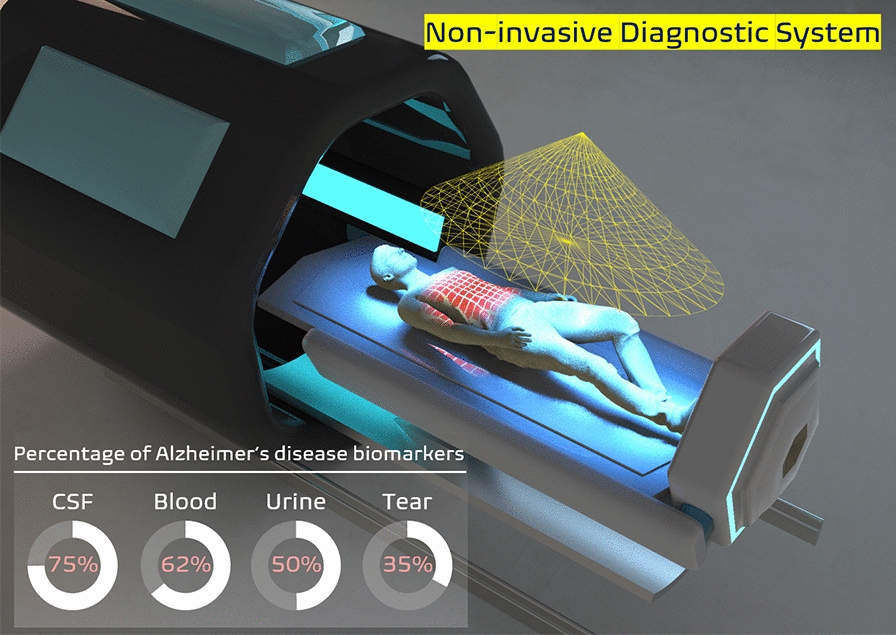

## Introduction

Globally, a large number of people with old age are affected by prolonged neurodegenerative diseases that can ultimately lead to Alzheimer’s disease (AD), the most prevailing type of dementia [1]. Biomarker-based detection can diagnose AD in an early stage. These biomarkers such as amyloid beta (Aβ) peptide, total tau protein (t-tau), phosphorylated tau protein (p-tau), and so on are well known as good indicators of AD [2]. Especially, Aβ peptide produced by amyloid precursor protein (APP) can be solubilized in cerebrospinal fluid (CSF) and blood plasma [3]. This biomarker enables high sensitivity and specificity in differentiating AD from other dementia. For this reason, various biosensing technologies are being developed using body-derived biosamples with solubilized biomarkers for early diagnose of AD.

Biomarkers present in various biosamples are key factors to identify early onset of AD. They are also helpful for the diagnosis, prognosis, and disease management process [4, 5]. These biomolecules indicate main aspects of pathogenesis such as neuronal and axonal degenerations [6, 7], neurofibrillary tangle (NFT) formation with p-tau [8, 9], and oligomerization of beta-amyloid to form amyloid plaques in the brain [10]. Aβ-peptides, t-tau, and p-tau proteins are the most common AD biomarkers. In 1984, a postmortem brain analysis indicated that Aβ-peptide was the primary component that triggered AD. Further research has added tau protein as a second key element for neurofibrillary tangles (NFT) and the pathological hallmark of AD. Later, amyloid precursor protein (APP) on chromosome 21 was identified as the cause of the formation of β-amyloid protein. APP has a direct relationship with Down syndrome (DS). It ultimately leads to Alzheimer’s disease [11]. Incessant research on AD has uncovered additional factors such as apolipoprotein-E (ApoE) [12] on chromosome 19, amyloid-β-derived diffusible ligands (ADDLs) [13], α-1-antitrypsin (AAT) [14], A disintegrin and metalloprotease 10 (ADAM10) [15], beta-secretase or beta-site APP cleaving enzyme I (BACE1) [16], and Alzheimer’s disease-associated neuronal thread protein (AD7c-NTP) [17].

The diagnosis of AD can be carried out using different biosamples from the body, such as cerebrospinal fluids (CSF), blood, urine, tears, sweat, and saliva [18, 19]. In majority of the cases, clinical diagnosis of AD has been carried out using brain-based biosamples (i.e., CSF) because they contain high concentrations of biomarkers [20–23]. CSF is an extracellular bio-fluid in the brain. It contains major AD biomarkers such as amyloid beta (Aβ) peptide and total tau protein (t-tau) along with phosphorylated tau protein (p-tau) [24]. Therefore, CSF is considered as a preferred biosample that can distinctly reflect pathological or biochemical changes in the brain as AD progresses [25]. However, the disadvantage of using CSF is that collecting it from the back bone of the body by lumbar puncture is invasive and uncomfortable. Such collection might induce transient back-pain and headache in patients [26–28]. As an alternative, blood-based biosensing is an attractive and practical strategy for the detection of AD biomarkers to overcome drawbacks of CSF collection [29, 30]. Blood sample can be used for diagnosis with a small volume. It contains Aβ-peptide, t-tau, p-tau, axonal neuron-specific protein neurofilament light (NFL), and other AD biomarkers [31]. Therefore, previous AD biosensor have mostly focused on effective detection and measurement of biomarkers from blood samples [32, 33]. A diagnostic method using blood sample is much easier than a CSF-based procedure. However, collecting blood samples is still invasive.

**Fig. 1:**
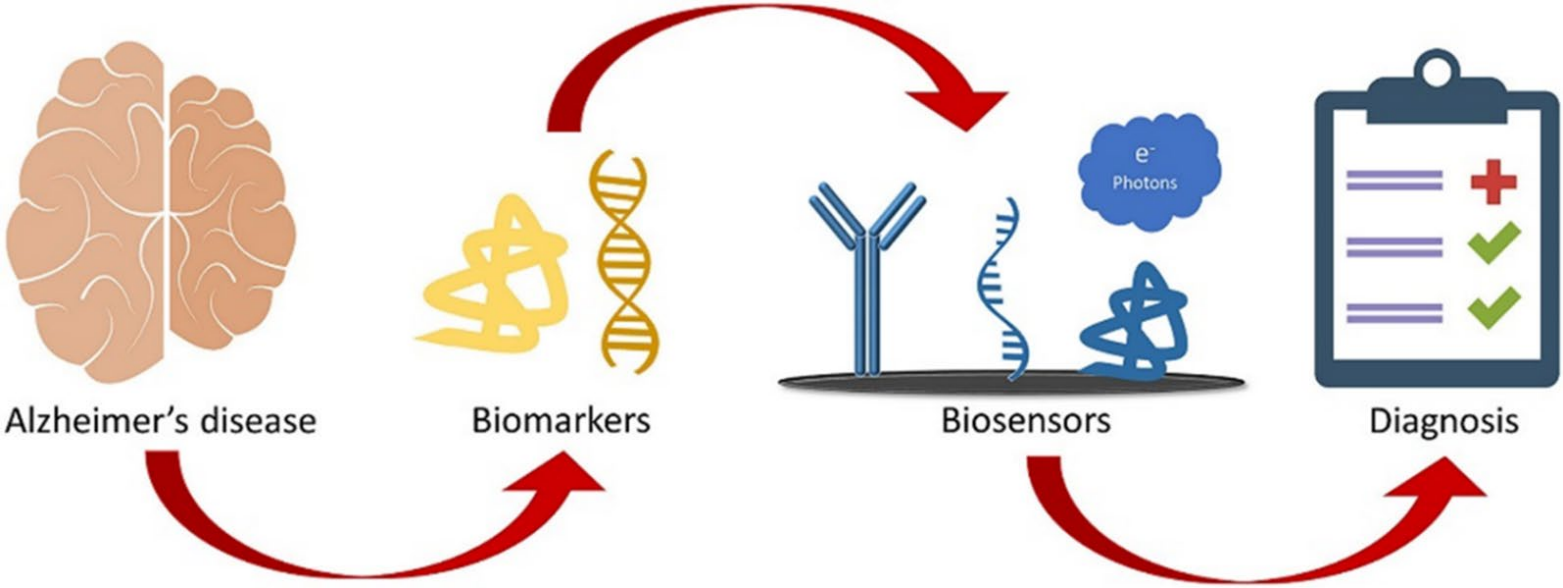
Schematic representation of AD biosensor for the detection of various biomarkers present in different body fluids. Reprinted with permission from ref. [34]

AD diagnosis would be more feasible to the general public if the detection of biomarkers can be achieved with low-cost and non-invasive techniques [34]. Non-invasive biosamples such as urine, tears, and sweat can be easily collected at the time of requirements to minimize disturbance to patients. They are attracting intensive research attention. However, since brain-released specific biomarkers are present at lower concentrations in non-invasive biosamples than in invasive samples (CSF and blood), an enhanced ultrasensitive detection of AD biomarkers using those non-invasive biosamples is required condition [35–40]. Urine, a non-invasive sample, is the product of blood filtration through the kidney. It contains different bio-components such as electrolytes, metabolites, and hormones. Different pathological evidences have indicated that a biomolecule 8-hydroxy-2-deoxyguanosine (8-OHdG) produced by oxidative damage of DNA is a significant biomarker in the urine of AD patients [41]. AD patients’ urine show higher levels of 8-OHdG than those of healthy individuals [42]. Similarly, the lacrimal apparatus produces tear, a watery fluid that oozes out from eyes. Tears contain different components such as electrolytes, lipids, and proteins that could be potentially used as disease indicators [43]. Many research and investigations are going on extensively to identify reliable relationships between tear biomolecules and human diseases [44]. Studies of tear biomarkers are shifting from traditional investigation approaches to more advanced technologies. Enzyme-linked immunosorbent assay (ELISA) and protein-chip arrays are some of commonly used modern techniques for the detection of AD biomarkers in tear samples [45, 46]. Human body produces a watery liquid that is well-known as sweat from the skin surface generally when the body faces an excessive physical exercise. It could be used as an important non-invasive biosample to monitor health condition of the body in a very easy way as it contains different types of electrolytes and biomolecules. For example, sodium ion present in sweat sample could be used to monitor and detect the disease progression of AD.

AD indicates that the appearance of neuropathological hallmarks in the body occurs more than one decade before the actual appearance of clinical symptoms [47, 48]. Advanced biosensor technology provokes the diagnosis of AD before the actual appearance of pathological symptoms. It plays a significant role in prompt management of the disease. The quality of the effective biosensors can be assessed with the reproducibility, accuracy, sensitivity, and stability of the sensor platform as well as its sensing results. Accuracy can be obtained by comparing the sensitivity of sensor platform with the gold standard method established for that particular biomarkers [49]. Contemporarily investigated and designed advanced biosensor platforms showed the improved accuracy, reproducibility, and stability for the detection of AD biomarkers from different body fluids samples. Therefore, the modern biosensor devices are designed to fulfil the demand of point-of-care guidelines with the enhanced accuracy performance [50]. Early detection of AD biomarkers using highly selective and ultrasensitive biosensors can be a significant step to alleviate diseases. Previously, the most commonly used ELISA method also known as INNOTEST assay has been applied for the detection of t-tau, p-tau, and Aβ42 in CSF samples [51, 52]. The use of capillary electrophoresis, western blotting, northern blotting, microRNA array technique, and real-time reverse transcription polymerase chain reaction is becoming popular for the detection of Aβ-peptides, tau proteins, and microRNA biomarkers [37]. An effective biosensor comprises different components successfully assembled for the ultrasensitive and highly selective detection of targeted biomarker analyte from biosamples as shown in Fig. 1, reprinted with permission from ref. [34]. With increasing prevalence of AD, there is a towering demand for a new approach of AD diagnosis using non-invasive methods. Biosensor techniques are highly effective and sensitive for real-time detection of biomarkers or biomolecules through an in vitro or in vivo process [53, 54]. Immuno-magnetic reduction (IMR) and/or single-molecule array (Simoa), mass spectrometry, and immunoassay tools are frequently used for the detection and measurement of biomarkers [55, 56]. Existing neuroimaging tools and neuropsychological tests are very expensive and out of reach for common people [57]. Therefore, cost-effective, miniaturized, and portable biosensors with easy handling procedure even by low-skilled general public show great value for the diagnosis and management of AD [58, 59].

This review highlights development, challenges, and future directions of biosensors using both invasive and non-invasive biosamples for early diagnosis of AD biomarkers. We provide the change of analytical performance criteria related to the progress of biosensors depending on biomarker detection scale (such as femto, nano, and micro) using different biosample types. It might be helpful to derive optimal ideas on the design, fabrication, and testing strategy for ultrasensitive biosensors to facilitate early detection of AD biomarkers in different biosamples.

### Detection of AD biomarkers in biosamples

**Fig. 2:**
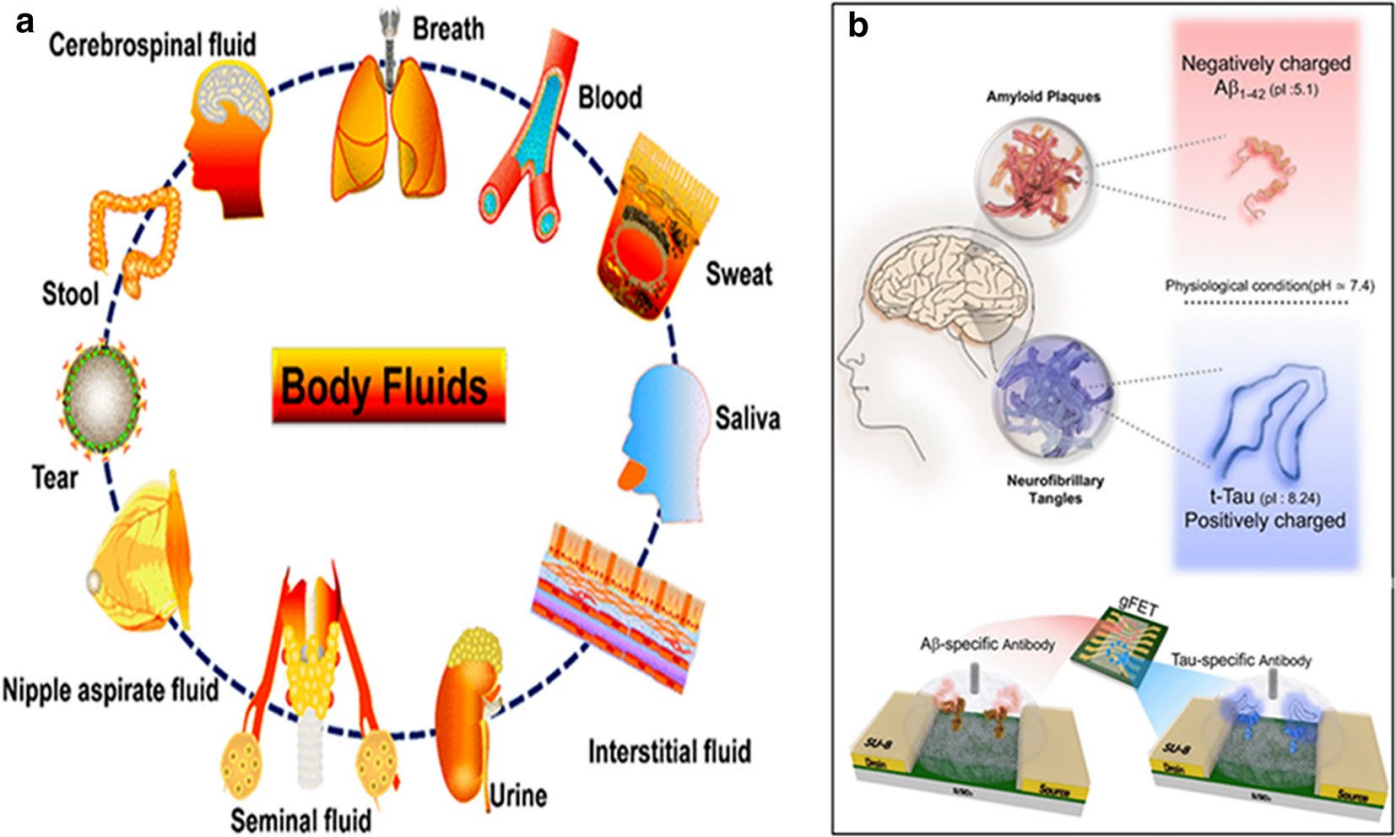
Body fluids and AD biomarkers sensing technique: **a** Sources of different biosamples from human body. Reprinted with permission from ref. [19], and **b** Multiplex detection of Aβ42 peptide and t-Tau protein using graphene-based field effect transistor (gFET) biosensor. Reprinted with permission from ref. [67]

There are various types of body fluids (blood, breath, CSF, interstitial fluid, nipple aspirate fluid, saliva, seminal fluid, stool, sweat, tear, and urine) as shown in Fig. 2A that may be used for the detection of AD biomarkers. Among these diverse body fluids, several specific body fluids containing AD biomarkers can be used to determine the progression of AD along with the advancement of biosensor technology.

Medical history has shown that brain-based components, mainly brain tissue, brain extract, and CSF, can be used at the very initial time to diagnose and monitor neurodegenerative diseases [60]. CSF is one of important biosamples used to diagnose AD. It can function as an indicator of any pathological changes in and around the brain or neural tissues [61]. Roche Diagnostic center has developed with a fully automated, precise, accurate, reliable and reproducible next-generation Elecsys assay platform, Roche cobas e601 analyzer, for the detection of CSF Aβ42, t-tau, and p-tau [62, 63]. This is an electrochemiluminescence immunoassay with quantitative sandwich principle and possesses the total assay time within 18 minutes. The measurement of Aβ peptides in CSF of AD patients could be very useful for drug development and clinical control. Disruption and permeability of the blood-brain barrier (BBB) increase with age [64]. Similarly, neurodegenerative disorders allow the infiltration of short peptide biomolecules such as Aβ peptides from the brain to the blood [65]. Therefore, blood samples also show the presence of important biomarkers, including Aβ peptides, t-tau, p-tau, and NFL (Fig. 2B). These Aβ peptide, t-tau protein, and p-tau proteins are the most promising biomarkers for the diagnosis of AD [66].

However, collecting CSF and blood samples are invasive mode, the recent studies have focused on early diagnosis of AD using non-invasive biosamples containing AD biomarkers. Urine is a potential biosample for the detection of AD biomarkers. The production of some specific biomarkers or biomolecules from the metabolism of proteins and lipids may indicate health condition of a person [68]. Hartmann et al. have used urine to detect AD biomarkers. Significant urine biomarkers include 8,12-iso-iPF_2α_-VI, total free amino acids, glycine, and 8-OHdG, with detected accuracy of 90 % [30, 69]. Isoprostane 8,12-iso-iPF_2α_-VI is a product of lipid peroxidation. Its concentration is increased in AD patients than in healthy controls [70]. Another study has reported that the level of an inhibitory neurotransmitter in the spinal cord, a free amino acid known as glycine, increases in AD patients [71]. Additionally, 8-OHdG has been reported as a critical AD biomarker in urine of patients. It is produced from the breakdown of guanine base of DNA by excited oxygen species [42, 72]. AD can also affect the entire visual capacity of a patient along with visual pathways and the visual cortex due to the deposition of Aβ peptides on walls of lens and retina [73, 74]. Therefore, tear is another important biosample containing relatively high protein contents [75] that could be used for the diagnosis of AD. Moreover, biomarkers such as lipocalin-1, dermcidin, lysozyme-C, lactotransferrin, defensins, LL-37 cathelicidin, and lacritin, could be used to detect AD by monitoring their concentrations and compositions in tear samples [76, 77]. Significant AD biomarkers could be obtained from sweat to monitor health conditions of patients [78]. Sweat, an epidermal bio-fluid, contains different biomolecules and/or electrolytes due to their passage under hydrostatic or osmotic pressure from blood or other body fluids [79–83]. It is seldom used as a biosample for the diagnosis of AD due to a very dilute aqueous solution with very low concentrations of proteomics and biomolecules associated with AD. Thus, a biosensor with special ultrasensitive capacity is required to detect different biomarkers in sweat [84, 85]. Saliva is another potential biosamples containing different complex secretions and is considered to have direct relation with nervous system could show an important source of biomarkers for different types of nervous disorders [86, 87]. It contains various biomolecules passed from blood due to microfiltration or active transport. Therefore, saliva might be a promising source for the early and accurate detection of AD biomarkers [88, 89]. Primarily, t-tau and p-tau are studied for the diagnosis of AD but another significant biomarker acetylecholinsterase, a type-B carboxylesterase enzyme is also studied as remarkable AD biomarkers which is associated with low concentration in AD patients [90]. Additionally, brain shows the presence of lactoferrin which substantially increases in AD patients. It is also correlated with the AD pathogenesis because of the direct relation with microgila of brain, amyloid beta plaques, and NFT of patients [91, 92]. Although, there are various AD related biomolecules in secretions of salivary glands, the conclusiveness of the results is still limited due to the lack of proper and sufficient standardization [93]. Similarly, the nasal secretions and olfactory fluids might also be considered as the promising non-invasive biosamples for the early detection of AD biomarkers [94–96].

AD-related biomarkers can be incorporated for diagnosing early onset and progression of AD. Tau or τ-proteins are a group of six highly soluble protein isoforms with lengths ranging from 352 to 441 amino acids. They are produced by alternative splicing of microtubule-associated protein tau (MAPT), a heat-stable protein [97, 98]. Neurons of the central nervous system (CNS) are major sources of tau proteins [99]. A number of phosphorylation sites are present on tau protein. They are used to control the binding capacity of tau protein with different microtubule structures [97]. There is a dynamic equilibrium between phosphorylation and dephosphorylation of tau protein within the cell body [100]. Initially, dephosphorylated tau protein shows extensive associated forms of microtubules. With the initiation of phosphorylation, it begins to disassemble to form microtubule units [101]. Pathological hallmarks in the brain show the presence of excessive hyperphosphorylation of tau which forms insoluble neurofibrillary tangles (NFTs) and results in synaptic dysfunctions and neural cell damage [102]. The hyperphosphorylated form of tau is an insoluble intra-neuronal collection of proteins. This is the major element that forms NFTs. It is regarded as a key feature of AD [103].

Serial cleavage of APP results in the formation of Aβ. During cleavage, it produces peptides with chain lengths of 36 to 43 amino acids. Aβ-peptide with aggregated fibrillary networks of plaques is considered the major pathological marker for AD. These plaques contain Aβ protein as a major component. Peptides having 40 and 42 amino acid units are indicated as Aβ40 and Aβ42, respectively [104]. Due to the tendency of higher aggregation, longer amyloid peptide (i.e. Aβ42) undergoes oligomerization to produce soluble dimers or higher-order oligomers [105]. Continued aggregation of higher-order oligomers forms Aβ fibrils. This is considered as a key factor for neurotoxicity caused by the production of toxic amyloid plaques, ultimately leading to neuronal cell death [106–108]. According to the amyloid hypothesis, Aβ is regarded as a promising biomarker for AD [109, 110].

Apolipoprotein E (ApoE) is a polymorphic allele and the main genetic indicator of AD. ApoE is an important cholesterol carrier that supports lipid transport and repairs brain injury. The presence of ApoE ɛ4 alleles in bio-fluids is strictly associated with genetic risk factors involved in the emergence of cerebral amyloid angiopathy and age-related cognitive impairments that cause late onset of AD [111]. ApoE joins with a number of receptors on the cell surface to deliver lipids and hydrophobic Aβ-peptides, thus introducing toxicity, causing synaptic dysfunction, and resulting in the deterioration or death of neurons that finally leads to AD. It induces the risk of appearance of β-amyloid senile plaques and neuritic plaques. Therefore, ApoE ɛ4 alleles are considered as important indicators for the detection and prognosis of AD.

Along with aforementioned biomarkers, other biomarkers of different categories, including genetic biomarkers, neuroimaging biomarkers, clinical biomarkers, and biochemical biomarkers, have been reported. For example, Aβ peptide-derived diffusion ligands (ADDLs) [112], APP [113], BACE1 [114], α-1-antitrypsin (AAT) [115], Alzheimer-associated neuronal thread protein (AD7c-NTP) [116], and NFL [117] are some significant AD biomarkers that have attracted tremendous research interest. Some biomolecules such as transthyretin [118], retinol-binding protein (RBP), β2-microglobulin [119], ApoA1 (apolipoprotein A1) [120, 121], granin-like neuroendocrine precursor [122], pigment epithelium-derived factor (PEDF) [123], and haptoglobin [124] are also associated with clinical diagnosis of AD [125, 126].

### CSF-based small biosensors

AD biomarkers from CSF sample could offer reliable pathological evidence to diagnose AD prior to the onset of pathological hallmarks. The first measurement of Aβ peptides from CSF was recorded by Tamaoka et al. in 1999 [127] and later in 2001. The t-tau protein was also determined in a CSF sample from a patient with Down’s Syndrome (DS) [128]. A reverse relationship in the concentration of Aβ42 peptides against t-tau and p-tau181 proteins indicates specific pathological conditions of AD patients compared to mild cognitive impairment (MCI) patients and normal controls. For example, decreased concentration of Aβ peptide and increased concentration of tau proteins in CSF samples are associated with amyloidosis and the risk of AD progression. Clinical reports have shown the presence of low concentration of Aβ42 but high concentrations of t-tau and p-tau in biosamples of AD patients than normal controls. These unusual proportions of biomarkers in CSF samples indicate the development of Aβ plaques that can ultimately result in neuronal damage, neural degeneration, and formation of neocortical neurofibrillary tangles [129]. An increased level of NFL chain in CSF has been reported to be correlated with the appearance of AD [130]. Recently, Kang et al. developed the AuNPs loaded sensor platform to apply the localized surface plasmon resonance (LSPR) technique to detect Apolipoprotein (ApoE4) [131]. Table 1 shows results of comparison of analytical performances of different types of femto-, nano-, and micro-biosensors used to detect AD biomarkers from CSF samples.

**Table 1:** Analytical performances of CSF-based biosensors

Sensor type		Target	Analytical method	Detection range	Limit of detection	Refs.
Femto- sensors	Magnetic nitrogen-doped graphene (MNG) immobilized with Aβ antibody (MNG-Aβab) on Au electrode	Aβ42	Cyclic voltammetry (CV) and differential potential voltammetry (DPV)	5000 fg/mL- 800 pg/mL	5000 fg/mL	[132]
	Ru(bpy)_3_^2+^/Zinc oxalate metal-organic framework (MOF)	Aβ	Electrochemical impedance spectroscopy (EIS)	100 fg/mL–50 ng/mL	13.8 fg/mL	[133]
	Cellular prion protein (PrP^c^) with a layer of poly(3,4-ethylene dioxythiophene (PEDOT) embedded AuNPs	AβO	EIS	0.1 fM–10^4^ nM	0.1 fM	[134]
Nano- sensors	Raman dye-coated polyA aptamer-AuNPs (PAapt-AuNPs)	Aβ42 and Tau	Surface-enhanced Raman scattering (SERS)	1 × 10^− 1^–1 × 10^4^ nM	3.7 × 10^− 2^ nM and 4.2 × 10^− 4^ pM	[135]
	Anti-Tau modified screen printed carbon electrode (SPCE) with graphene oxide (GO) and amine functionalized trimethylolpropane tris poly(propylleneglycol) (pPG)	Tau	DPV and EIS	0.5–15.1 nM	0.15 nM	[136]
	2,2’-azino-bis(3-ethylenebenzothiazoline-6-sulfonic acid-polydiallylmethylammonium chloride (ABTS-PDDA) bi-functionalized with SWCNTs loaded with neurokinin B	Aβ42	DPV	1 ng-0.38 µg/mL	0.5 ng/mL	[137]
	AuNPs deposited on polyethylene terephthalate (PET) film by Langmuir-Blodgett (LB) method	Aβ42	Localized surface plasmon resonance (LSPR)	-	0.001 ng/mL	[138]
	Au disc coated with polypyrrol-2-COOH film immobilized NH_2_-terminated PrP^c^	AβO	EIS	10^− 7^–0 nM	10^− 7^ nM	[139]
	Graphene oxide-based platform immobilized with AβO antibody	AβO	Fluorescence	10 nM–2 µM	1 nM	[140]
Micro-sensors	Tau protein-based Au disk electrode	Tau441	CV and EIS	0.1–1.0 µM	0.2 µM	[141]
	AD7c-NTP antibodies immobilized sandwich immunoassay	AD7c-NTP	ELISA	-	0.0092 µg/mL	[142]
	96 well microtiter plates coated with monoclonal antibody (N3I4)	AD7c-NPT	ELISA	3 × 10^− 5^-5 × 10^− 5^ µg/mL	0.002 µg/mL	[143]
	Reduced graphene oxide (RGO)-based enzyme-modified field-effect transistor (RGO-EnFET)	Acetylcholine (ACh)	Electrical conductance measurement	1 µM–10 mM	1 µM	[144]

#### Femto-biosensors to detect CSF-based biomarkers

**Fig. 3:**
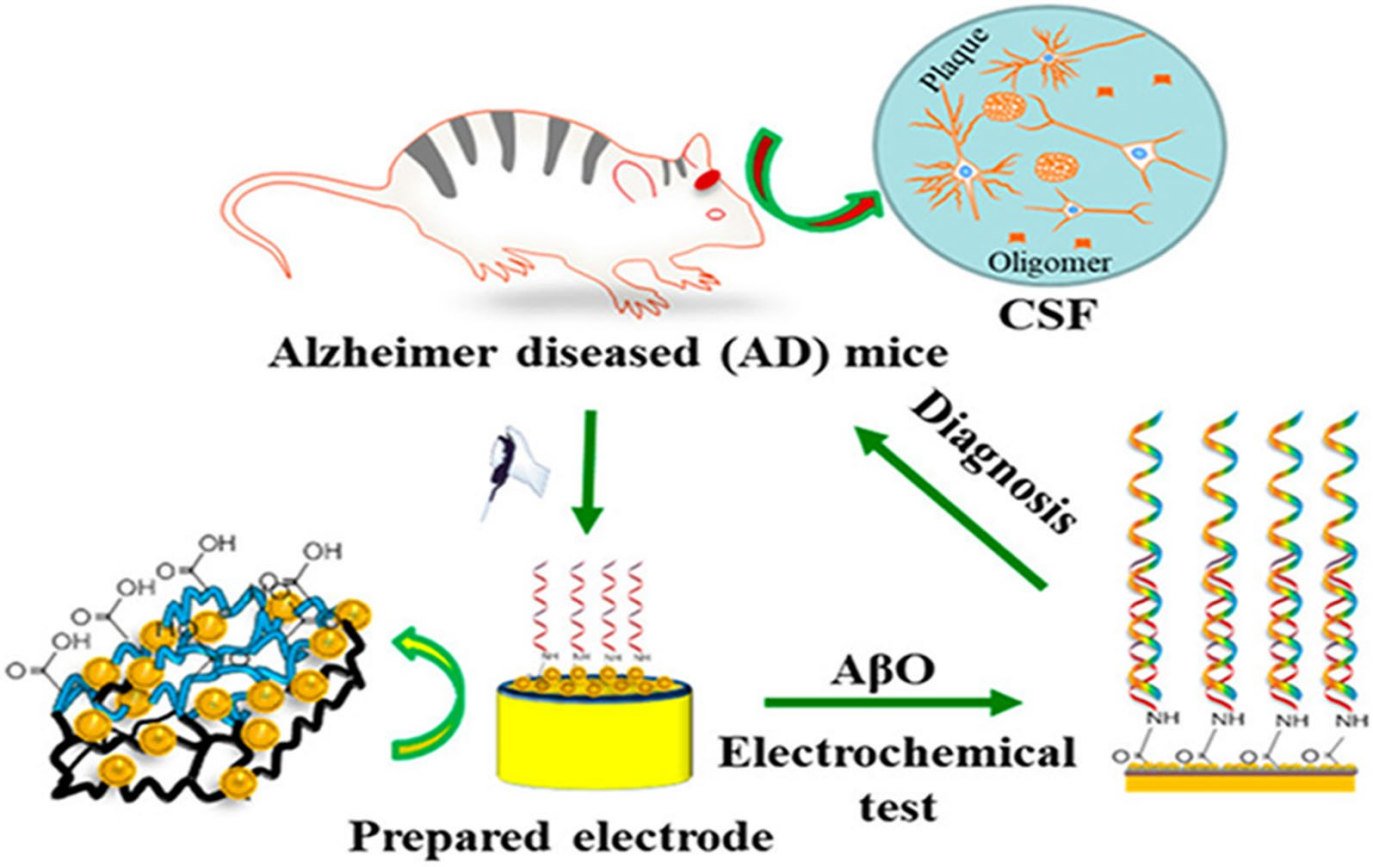
Ultrasensitive detection of amyloid-beta-oligomers in mice CSF using a poly(3,4-ethylene dioxythiophene) (PEDOT)-embedded Au nanoelectrode. Reprinted with permission from ref. [134]

Femto-level detection of biomarkers is required to facilitate the diagnosis of AD when concentrations of biomarkers are very low in biosamples. Li et al. have fabricated a magnetic nitrogen-doped graphene (MNG) immobilized with anti-Aβ antibody (MNG-Aβab)-modified Au electrode. They used cyclic voltammetry (CV) and differential pulse voltammetry (DPV) techniques for the electrochemical measurements. Such MNG-Aβab-modified AuSPE biosensor was highly effective for the detection of Aβ42 peptide with a linear range of 5000 fg/mL to 800 pg/mL and a limit of detection (LOD) of 5000 fg/mL [132]. Three-dimensional zinc oxalate-metal organic frameworks Ru(bpy)_3_^2+^/Zinc oxalate 3D MOFs have been fabricated for femto molar detection of Aβ42 peptide from artificial CSF (aCSF) and BSA solutions using electrochemical impedance spectroscopy (EIS) technique, with a linear range of detection from 100 fg/mL to 50 ng/mL and an LOD of 13.8 fg/mL [133]. Furthermore, an ultrasensitive electrochemical biosensor with cellular prion protein (PrP^C^) bio-receptor combined with poly(thiophene-3-acetic acid) as a transducer has been fabricated for the detection of amyloid beta oligomer (AβO) using EIS technique as shown in Fig. 3. Qin et al. utilized mice brain-extracted CSF and obtained a wide range of detection within a subfemtomolar scale of 10^− 8^ to 10^4^ nM and an LOD of 10^− 2^ fM [134]. Thus, femto-biosensors using CSF samples are very significant for the detection of ultralow concentration of AD biomarkers for early diagnosis of diseases.

#### Nano-biosensors to detect CSF-based biomarkers

**Fig. 4:**
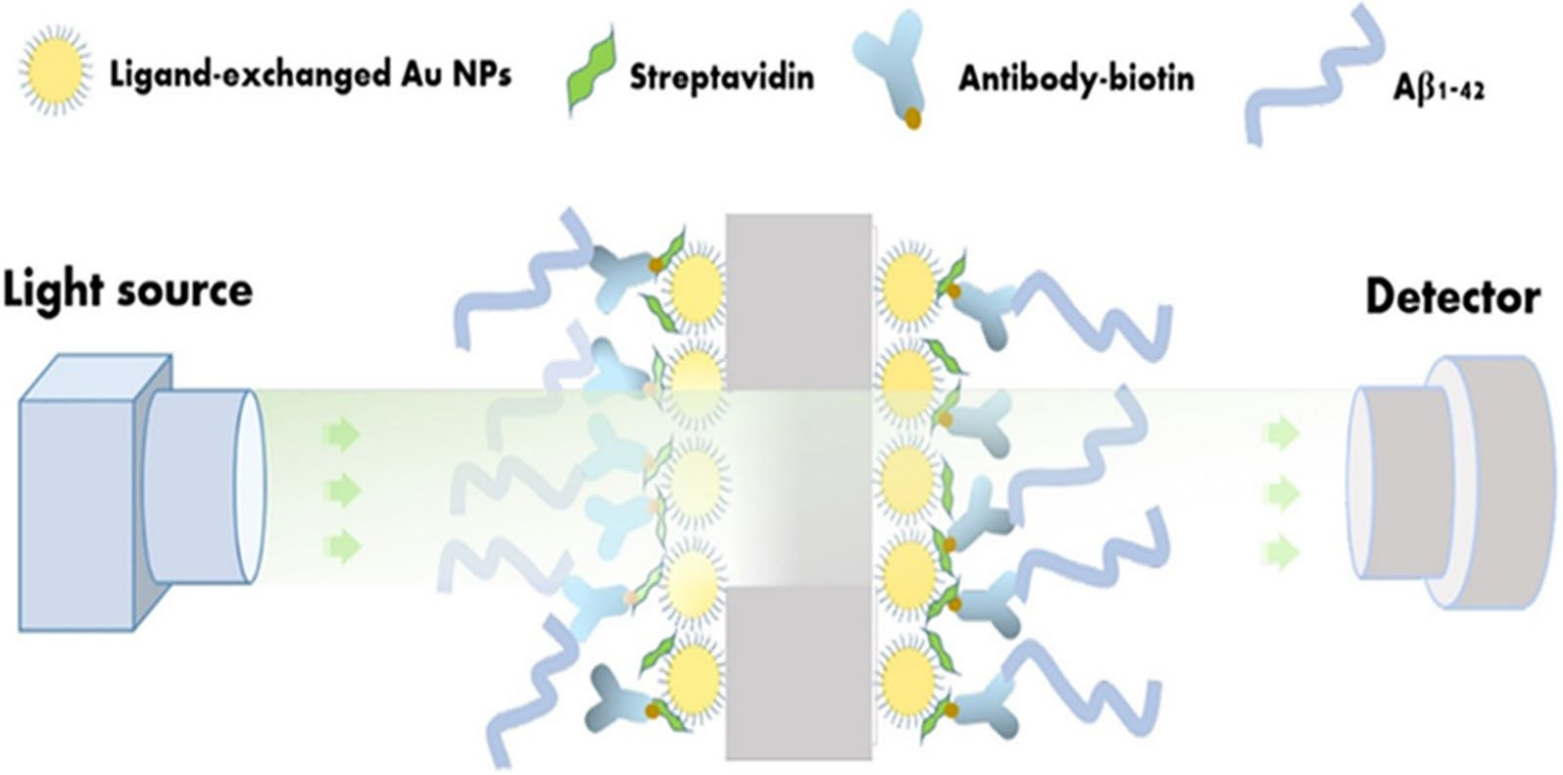
Ultrasensitive detection of amyloid-beta-oligomers in mice CSF using a poly(3,4-ethylene dioxythiophene) (PEDOT)-embedded Au nanoelectrode. Reprinted with permission from ref. [138]

Majority of AD biomarkers are detected in the nanoscale range. With improved technologies, some researchers have developed a wide range of biosensors for nanoscale detection of AD biomarkers. A surface-enhanced Raman scattering (SERS) biosensor platform has been fabricated by Zhang et al. using Raman dye-coated polyA aptamer-AuNPs (PAapt-AuNPs) and SERS technique for the detection of Aβ42 oligomers and tau protein with a wide linear range of detection 1 × 10^− 1^ to 1 × 10^4^ nM and an LOD of 3.7 × 10^− 2^ nM for Aβ42 and 4.2 × 10^− 4^ pM for tau protein [135]. Localized surface plasmon resonance (LSPR) technique was utilized for the detection of amyloid beta (1–42) peptide as shown in Fig. 4, using an optical sensor fabricated using ligand-exchange AuNPs deposited on polyethylene terephthalate (PET) film by Langmuir-Blodgett (LB) method. This sensor detected Aβ42 peptide with the limit of detection as 0.001 ng/mL [138]. Similarly, an antibody immobilized graphene oxide biosensor conjugated with dendrimer-PbS/CdS electrochemical nano-platform has been prepared for the detection of tau protein. This immunosensor used DPV and electrochemical impedance spectrometry (EIS) techniques and established a linear range of detection as 0.5–15.1 nM with an LOD of 0.15 nM [136]. Yu et al. have designed a single ratio-metric electrochemical biosensor of 2,2’-azinobis-3-ethylbenzothiazoline-6-sulphonate (ABTS) and poly(diallyldimethylammonium chloride) (PDDA) bi-functionalized single-walled carbon nanotubes (SWNTs) composite to detect Aβ42 from the hippocampus of AD rats. This biosensor applied DPV method to detect Aβ42 with an LOD of 0.5 ng/mL and a linear range of detection as 1 ng/mL to 3.08 µg/mL [137]. Qin et al. have prepared cellular prion protein and poly(pyrrole-2-carboxylic acid) immobilized electrode to monitor amyloid beta oligomers for early diagnosis of AD biomarkers. They used mice CSF samples for the detection of oligomers using cyclic voltammetry (CV) and EIS technique, showing a limit of detection of 10^− 7^ nM [139]. Aβ oligomers (AβOs) could be detected from artificial CSF samples using a graphene oxide-based biosensor platform for selective capturing of target oligomers with a fluorescence method. This tool was successful for the detection of oligomers in a wide linear range of concentrations from 10 nM to 2 µM with a limit of detection of 1 nM [140].

#### Micro-biosensors to detect CSF-based biomarkers

For micro-scale detection of AD biomarkers, a researcher group has fabricated anti-tau protein modified Au disk electrode for the detection of tau protein from CSF biosamples using CV and EIS technique. They observed a linear range of detection of 0.1–1.0 µM and an LOD of 0.2 µM [141]. Additionally, the measurement of AD7c-NTP showed diagnostic features of AD patients. A group of researchers have used ELISA technique-based biosensors for the detection of AD7c-NTP biomarker from postmortem human CSF samples. The device could successfully measure levels of the AD7c-NTP biomarker with a limit of detection of 0.0092 µg/mL in Alzheimer’s patients, higher than that of normal controls (0.0016 µg/mL) [142]. Ghanbari et al. have designed a sandwich-type enzyme immunoassay to measure levels of AD7c-NTP biomarker in CSF samples of AD patients. This sandwich assay was able to detect the biomarker at a linear range of detection of 3 × 10^− 5^ to 5 × 10^− 5^ µg/mL with a limit of detection of 0.002 µg/mL [143]. CSF is supposed to be a typical biosample for leukocytes trafficking into the CNS [145]. An enzyme-modified graphene-based transistor biosensor platform has been fabricated to detect acetylcholine (Ach) and monitor drug delivery. This transistor biosensor could measure Ach in a linear range of 1 µM to 10 mM using the electrical conductance method [144].

### Blood-based small biosensors

Blood is a popular biosample for in vitro diagnosis of diverse diseases. Although the blood-brain-barrier (BBB) separates the blood stream from the CSF, brain-derived proteins in a low quantity are selectively exchanged between these two bio-fluids. For this reason, blood sample can also be used exclusively for studying brain-related pathologies. In contrast to CSF, blood shows more complex compositions because of the presence of different biomolecules such as glucose, lipids, creatinine, hormones, antibodies, proteins, and cytokines. These interfering elements hinder biomarker detection using traditional detection platforms. Furthermore, low concentrations of brain-associated biomarkers in blood samples demand highly selective and sensitive tools for biomarker detection. Different blood-based biosensors with their detection targets, ranges of detection, and limits of detection are summarized in Table 2.

**Table 2:** Analytical performances of blood-based biosensors

Sensor type		Target	Analytical method	Detection range	Limit of detection	Refs.
Femto- sensors	Electrochemically reduced graphene oxide (ERGO) and AuNWs modified screen printed carbon electrode (SPCE)	microRNA137	Cyclic voltammetry (CV) and electrochemical impedance spectroscopy (EIS)	5.0–750.0 fM	1.7 fM	[33]
	Densely aligned CNTs on Si wafer	t-Tau P-tau181 Aβ40 and Aβ42	Electrochemical resistance measurement	10–10^6^ fM	2.45 fM 2.72 fM 2.2 fM and 2.13 fM	[146]
	Antibody immobilized 3-mercaptopropionic acid (MPA) and thiol modified polyethylene glycol (PEG-SH) on AuNPs screen printed carbon electrode (SPCE)	Aβ42	Deferential potential voltammetry (DPV)	100 fM–25 pM	100 fM	[147]
	Hybrid graphene coated with HS-PEG modified by antibody	Aβ and Tau	Surface enhanced Raman spectroscopy (SERS)	–	100 fg/mL	[148]
	Interdigitated microelectrode (IME) on polydimethylsiloxane (PDMS) microchannel with Ti sputtered over Pt electrode on Si-wafer	Aβ	EIS	1 ng/mL − 100 fg/mL	100 fg/mL	[149]
	Multi-walled carbon nanotubes (MWCNTs) and reduced graphene oxide-chitosan (RGO-CS) modified with AuNPs	t-Tau441	DPV	0.5–80 fM	0.46 fM	[150]
	Iron oxyhydroxide (FeOOH) conjugated Mo-doped BiVO_4_ modified by tau antibody	Tau	EIS	–	1.59 fM	[151]
	Poly(diallyldimethylammonium chloride) (PDDA) and AuNP bilayer electrode	ADAM10	CV	5.6-1388.9 fg/mL	0.35 fg/mL	[152]
	DNAaptamer/antibody sandwich assay	Tau381	Surface plasmon resonance (SPR)	2–80 pM	10 fM	[153]
	Gold-nanochip with 11-mercaptoundecanoic acid (11-MUA) monolayer coupled with tau antibody and antiAAT	Tau381	SPR	–	~ 39 fM	[154]
	Ceria doped ZnO NFs-based sensor	Aβ	ECL	80 fg/mL–100 ng/mL	52 fg/mL	[155]
	Aptamer-antibody sandwich assay with cysteamine-stabilized AuNPs	Tau381	DPV	500 − 10^4^ fM	420 fM	[156]
	Anti-tau functionalized hybrid magnetic NPs (MNP) with AuNPs	Tau	SERS	25 fM–500 nM	5 fM	[157]
	A shape code type AuNPs immobilized with antibody	Aβ40, Aβ42, and tau protein	localized surface plasmon resonance (LSPR)	1 × 10^1^ – 1 × 10^8^ fM	34.9 fM, 26 fM, and 23.6 fM	[158]
	AuNPs-based biochip conjugated with Aβantibody	Aβ42	SPR	1 µg/mL–10 fg/mL	10 fg/mL	[159]
	Four-electrode electrochemical biosensor with antibody-antigen complexes on Au electrode coated with protein G	2N4R tau protein	CV and EIS	10^− 4^–10 nM	30 fM	[160]
	Indium tin oxide (ITO) coated by 3-aminopropyltrimethoxysilane (APTMS) and glutaraldehyde (GA) conjugated with AuNPs	ApoE	SPR	1.0 pg/mL-10 ng/mL	420 fg/mL	[161]
	Ion concentration polarization-interdigitated microelectrode (ICP-IME)-based microfluidic chip	Aβ-peptide	Impedance measurement	1 ng/mL-1 pg/mL	8.15 fM	[162]
	A nanoplasmonic biosensor of Au nanorods with chaotic agent for the ultrasensitive detection of AD biomarkers		Localized surface plasmon resonance (LSPR)	102–108 fM	10 fM	[163]
	Photolithographically patterned electrodes with amino groups for reduced graphene oxide-based field effect transistor (RGO-FET)	Aβ	EIS	1 fM-100 pM	1 fM	[164]
Nano-sensors	Disposable label-free screen printed chip with self-assembled monolayer (SAM) and AuNPs	Aβ42	EIS	2.04 µM-2.65 nM	0.57 nM	[11]
	CdSe@ZnS quantum dots (QDs) labeled on polydimethylsiloxane-polycarbonate (PDMS-PC) microfluidic chip with integrated screen printed electrode (SPE)	ApoE	Square wave anodic stripping voltammetry (SWASV)	10–200 ng/mL	12.5 ng/mL	[165]
	Alkaline phosphatase (ALP) integrated onto hydroxyapatite (HAP) probe with molybdate as signal amplifier	BACE1	CV and Square wave voltammograms (SWVs)	5-150 nM	10 nM	[166]
Micro- sensors	Streptavidin interface probe sensor	Tau441	Optical Biotinylated interferometry (BLI)	2–55 nM	6.7 nM	[167]
	Fern leaves-like gold nanostructure, modified into RNA aptamer sensor	Aβ	DPV	0.002–1.28 ng/mL	0.4 pg/mL	[168]
	AuNPs coated with N-and C-terminal of dual antibody AuNPs@C/N-Ab(1–42)	Aβ42	Colorimetry	7.5–350 nM	2.3 nM	[169]
	Gold-nanochip with monolayer of 11- mercaptoundecanoic acid (11-MUA) coupled with antiAAT for the detection of Alpha-1 antitrypsin (AAT)	Alpha-1 antitrypsin (AAT)	SPR	~ 10–200 nM	~ 65 µM	[154]
	Self-assembled monolayers (SAM) of 3-mercaptopropionic acid (MPA) and thiol group over Au sputtered on polyethylene terephthalate (PET) film with Aβab	Aβ42	DPV	0.0675-0.5 µg/mL	-	[170]
	Label-free antibody modified Au electrode	Aβ1–16	Total internal reflection ellipsometry (TIRE)	0.5 × 10^− 4^ g/mL-5 µg/mL	0.5 × 10^− 4^ µg/mL	[171]
	Anti-tau antibodies coated on polycrystalline Au surface	Tau441	CV and EIS	1-100 µg/mL	10 µg/mL	[172]

#### Femto-biosensors to detect blood-based biomarkers

**Fig. 5:**
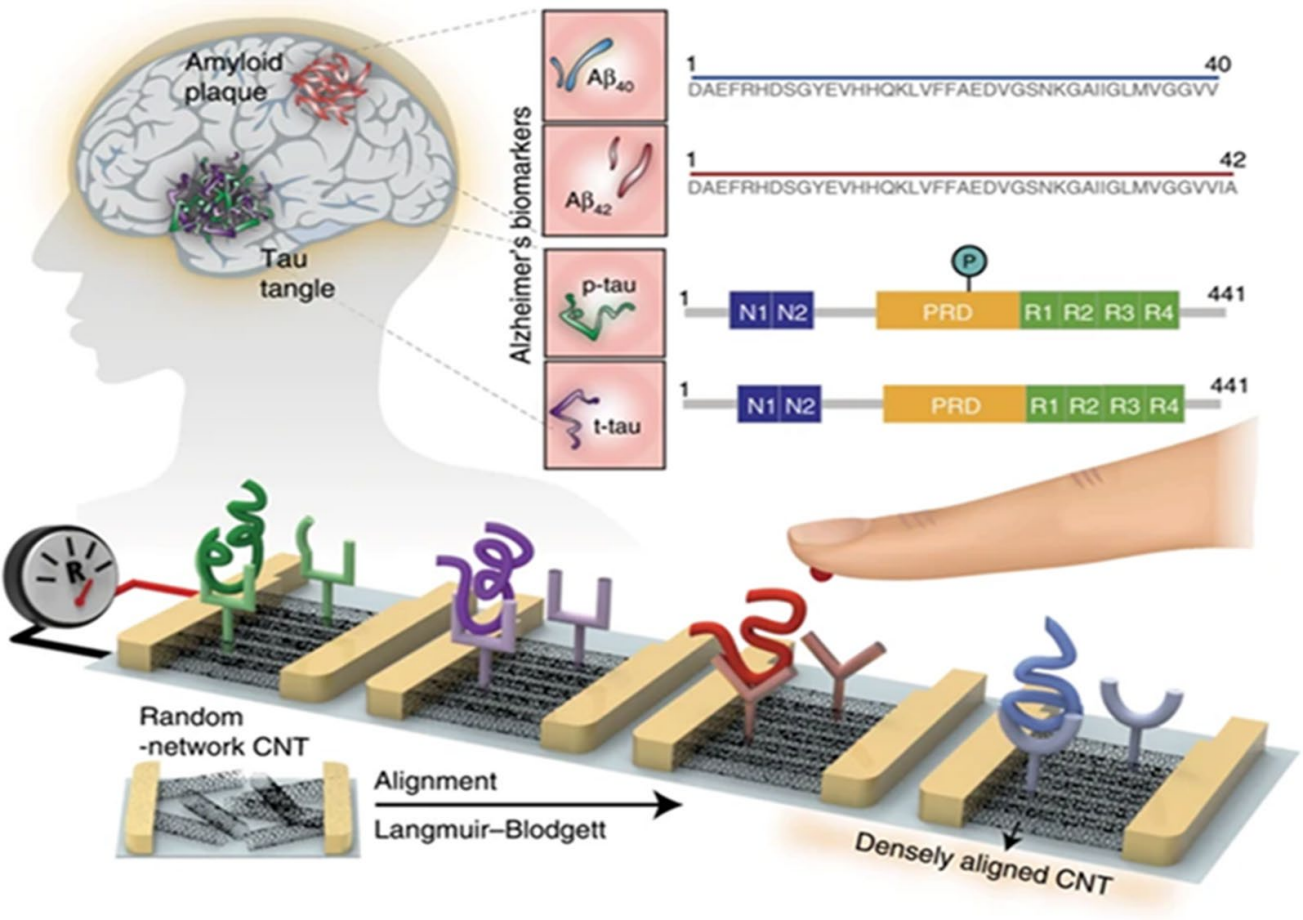
Thin film biosensor with densely aligned SWNTs for the multiplex detection of Aβ-peptide from blood. Reprinted with permission from ref. [146]

Electrochemically reduced graphene oxide (ERGO) and Au nanowires (AuNWs) have been used for the fabrication of screen printed carbon electrode (SPCE) with doxorubicin (Dox) and applied for the early detection of serum microRNA137 by CV and EIS techniques. Its linear range of detection was 5.0-750.0 fM and its LOD was 1.7 fM [33]. Kim et al. have successfully designed a closely packed and highly aligned CNT sensor array for multiplexed sensing of core biomarkers as shown in Fig. 5. This was used to measure levels of AD biomarkers using fluorescence and resistance measurement techniques, showing LOD values of 2.45, 2.72, 2.20, and 2.13 fM/mL for t-tau, p-tau181, Aβ42, and Aβ40, respectively [146]. An electrochemical immunoassay has been developed by Diba et al. from sandwich immunoassay fabricated with an antibody coated on a monolayer obtained by mixing 3-mercaptopropionic acid (MPA) and thiol modified polyethylene glycol (PEG-SH) localized on AuNPs over the screen printed carbon electrode (SPCE). Electrochemical impedance spectroscopy (EIS) has been applied for the detection of Aβ42 with an LOD of 100 fM and a linear range of detection from 0.1 fM to 25 fM [147]. Demeritte et al. have designed a label-free hybrid graphene oxide platform for simultaneous detection of Aβ42 and tau protein using surface enhanced Raman spectroscopy (SERS) technique. This biosensor device showed an LOD value of 100 fg/mL for Aβ42 and tau protein [148]. A shape code type AuNPs nanoplasmonic sensor was used for the detection of AD biomarkers from mimicked blood sample using localized surface plasmon resonance (LSPR). This sensor showed the LOD for Aβ40, Aβ42, and tau protein as 34.9 fM, 26 fM, and 23.6 fM respectively [158]. Similarly, Yoo et al. have designed a blood-based ultrasensitive label-free platform of integrated microelectrode (IME) sensor for the detection of Aβ-peptide using the EIS technique. It showed a detection range from 100 fg/mL to 1 ng/mL [149]. Further, MWCNTs, reduced graphene oxide (rGO), and chitosan (CS) have been applied for biosensor fabrication to detect tau441 protein with a linear range of detection of 0.5–80 fM and an LOD of 0.46 fM using the DVP technique [150]. Kim et al. have prepared an ultrasensitive biosensor using BiVO_4_-based photoelectrochemical (PEC) platform by incorporating molybdenum (Mo) dopant and iron oxyhydroxide (FeOOH) layer on the photoelectrode with horseradish peroxidase (HRP) as a signal amplifier to trigger the oxidation of 3,3’-diaminobenzidine (DAB) [151]. They used the EIS technique to measure tau in blood plasma and found an LOD of 1.59 fM.

Another biomarker ADAM10 can be measured with a disposable microfluidic platform using poly(diallyldimethyl ammonium chloride) (PDDA) and AuNPs bilayer electrode from human blood plasma. The CV technique has been applied for the detection of biomarker, showing a detection range of 5.6-1.38 × 10^3^ fg/mL and an LOD of 0.35 fg/mL [152]. Similarly, Kim et al. have prepared a DNA aptamer/antibody sandwich assay for tau381 detection using a multichannel surface plasmon resonance (SPR) platform. This sensor could detect tau381 with an LOD of 10 fM and a detection range of 2 to 80 pM using the SPR technique [153]. Au-functionalized monolayer of 11-mercaptoundecanoic acid (MUA) coupled with tau antibody has been used for an effective detection of tau381 by the SPR technique, showing an LOD of ~ 39 fM [154]. Luminol-based immunosensor with ceria doped ZnO nanoflower has been used for Aβ-peptide detection from PBS and human serum at an ultralow LOD of 52 fg/mL and a wide detection range from 80 fg/mL to 100 ng/mL using an electrochemiluminescence technique [155]. A nanoplasmonic biosensor using a chaotic agent was applied for the ultrasensitive detection of AD biomarkers from undiluted human plasma. This biosensor was able to detect the tau protein within the concentration range of 102 to 108 fM with the minimum detectable concentration as 100 fM using Localized surface plasmon resonance (LSPR) technique [163]. Additionally, tau-381 protein can be detected with cysteamine-stabilized AuNPs as signal amplifier employing DPV technique within a range of detection from 500 to 10^4^ fM and an LOD of 420 fM [156]. Anti-tau modified AuNPs have been functionalized with magnetic nanoparticles and used to detect tau381 by SERS technique. The device showed a linear range of detection of 25 fM to 500 nM with an LOD of 25 fM [157]. Scanning tunneling microscopy (STM)-based biochip has been prepared with AuNPs-Aβantibody conjugation for the detection of Aβ42 within a concentration range of 1 µg/mL to 10 fg/mL and an LOD of 10 fg/mL using the SPR technique [159]. A biosensor with four-electrode system has also been used for the detection of serum 2N4R tau protein applying CV and EIS techniques and obtained a linear range of detection of 10^− 4^–10 nM and an LOD of 30 fM [160]. Ren et al. have designed a colorimetric immunosensor using indium tin oxide (ITO) modified by 3-aminopropyltrimethoxysilane (APTMS) and glutaraldehyde (GA) conjugated with AuNPs-antibody using SPR and colorimetric technique for the detection of ApoE biomarker. The device could detect ApoE within a linear range of 1.0 pg/mL to 10 ng/mL and an LOD of 420 fg/mL [161]. Ion concentration polarization-interdigitated microelectrode (ICP-IME)-based microfluidic sensor has been prepared by Yoo et al. to measure Aβ-peptide using impedance measurement technique. It showed a linear detection range of 1 ng/mL–1 pg/mL and an LOD of 8.15 fM [162]. Kurkina et al. have used photo-lithographically patterned electrodes with amino groups to produce rGO-FET immunosensor for the detection of Aβ peptide from serum with an LOD of 1 fM and a linear range of 1 fM to 100 pM with the help of the EIS technique [164].

#### Nano-biosensors to detect blood-based biomarkers

A label-free impedimetric immunosensor on a carbon disposable electrochemical screen printed chip has been developed using self-assembled monolayer (SAM) and AuNPs for the measurement of Aβ42 [11]. The attractive disposable carbon chip could measure amyloid beta within a linear range of detection of 2.04 µM to 2.65 nM and an LOD of 0.57 nM. An on-chip magneto-immunoassay has been fabricated using cadmium selenide/zinc sulfide QDs. An immunocomplex device has been made with tosylated magnetic beads as a pre-concentration chamber on a polydimethylsiloxane (PDMS)-polycarbonate (PC) microfluidic chip with integrated SPE for the detection of biomarker ApoE using a square wave anodic striping voltammetry (SWASV) technique. The microchip could effectively measure the level of ApoE with a detection range of 10–200 ng/mL and an LOD as 12.5 ng/mL [165]. Qu et al. have designed alkaline phosphatase (ALP) with a redox-generating hydroxyapatite (HAP) probe for dual signal amplification and amyloid beta antibody immobilization on the surface of an electrode [166]. This sensing probe could detect BACE1 protease in human serum with a linear range of detection of 5-150 nM and an LOD of 10 nM with CVs and SWVs. Similarly, a simple bio-chip with a very easy handling process has been designed as a dip-and-read label-free optical aptasensor based on a biolayer interferometry (BLI) technique. It was developed for the detection of tau441 protein from buffer and serum solutions with a limit of detection of 6.7 nM and a linear range of detection of 2 nM to 55 nM [167]. This aptasensor was successfully developed with the BLI detection technique. Negahdary et al. have reported a linear range of 0.002 to 1.28 ng/mL and an LOD of 0.4 pg/mL for the detection of Aβ42. They have designed an interesting fern leaves-like gold nanostructure this is modified for RNA aptamer binding with Aβ that could be detected with a ferro/ferricyanide redox marker using DPV technique [168]. Another study has reported a sandwich immunosensor for the detection of Aβ42 based on dual antibody-modified AuNPs with a colorimetric technique. AuNPs were successfully coated with N- and C-terminal of antibodies, resulting in an aggregate of AuNPs@C/N-Ab(1–42). This device detected Aβ42 within a linear range of detection of 7.5 nM to 350 nM and an LOD of 2.3 nM [169].

#### Micro-biosensors to detect blood-based biomarkers

The micro-level sensing method for in vitro determination of Aβ42 peptides was carried out using the DPV technique. The sensor was fabricated with self-assembled monolayers (SAM) using 3-mercaptopropionic acid (MPA) to immobilize the thiol group on a 50 nm Au sputtered on polyethylene terephthalate (PET) substrate. The electrode was functionalized with Aβ-antibody (Aβ-ab) modified with 3-mercaptopropionic acid (MPA). It showed a linear range of detection of 0.0675 to 0.5 µg/mL [170]. Sometimes, the detection of smaller and water-soluble amyloid beta peptide molecules (Aβ1–16) might be a step forward in the development of biosensors with cost effective and rapid test techniques. One study has reported the use of total internal reflection ellipsometry (TIRE) immunoassay with DE2 antibodies for the detection of small water soluble amyloid beta peptides (Aβ1–16) [173]. Mustafa et al. have applied TIRE and quartz crystal microbalance (QCM) measurement techniques for the detection of Aβ1–16 and reported an LOD of 0.5 × 10^− 4^ µg/mL and a wide-range of linear concentrations from 0.5 × 10^− 4^ µg/mL to 5 µg/mL using antibody modified Au electrode [171]. Similarly, another study has reported an electrochemical immunosensor with a wide range of detection of 1 to 100 µg/mL and an LOD of 10 µg/mL for non-phosphorylated tau441 protein using tau-antibody coated on polycrystalline Au surfaces (Ab-Au) with CV and EIS techniques [172].

### Urine-based small biosensors

Urine is a popular sample with an easy mode of collection to diagnose diverse diseases. Table 3 presents different urine-based biosensors used for the detection of AD target molecules. Unlike other biosamples, the use of urine for the detection of AD biomarkers involves simple and standard procedures. The detection of AD biomarkers can be easily performed based on creatinine concentration [174]. The level of glycine, a type of amino acid, is increased in urine samples collected from AD patients [175].

**Table 3:** Analytical performances of urine-based biosensors

Sensor type		Target	Analytical method	Detection range	Limit of detection	Refs.
Femto- sensors	Fused silica column capillary with total length 42 cm and effective length 30 cm binding with anti-8-OHdG	8-OHdG	Capillary electrophoresis-laser-induced fluorescence (CE-LIF) detection	-	0.18 fM	[176]
Nano- sensors	Solid phase extraction (SPE) in C18/OH column and HPLC in 5µmC18 HPLC with 1 mL/min mobile phase flow rate	8-OHdG	High performance liquid chromatography-electrochemical detection (HPLC-ECD)	7.0–700 nM/L	0.35 nM/L	[42]
	Antibody modified sandwich immunoassay	AD7c-NTP	ELISA	-	0.48 ng/mL	[17]
	Polyvinylidene fluoride (PVDF) membrane with monoclonal antibody	Aβ	Western blots	-	0.04 ng/mL	[177]
	96-well microtiter plate coated with monoclonal antibody (N3I4)	AD7c-NTP	HPLC	-	2.5 ng/mL	[178]
Micro- sensors	Microtiter plate coated with bispecific receptor antibody having affinity for immunoglobulin G (IgG) and urinary NTP	AD7c-NTP	Enzyme-linked sandwich immunoassay (ELISA)	-	26.8 ± 9.4 µg/mL	[179]
	32-well microtiter plates immunoassay	NTP	ELISA	10–60 µg/mL	10 µg/mL	[180]
	Rabbit immunoglobulin G (IgG) coated immunosensor	AD7c-NTP	ELISA	-	32.76 ± 9.0 µg/mL	[181]
	Electro-polymerized Poly(neutral red) film modified with Fe_2_O_3_ magnetic nanoparticles (MNPs) embedded with acetylcholinesterase (AChE) using glutaraldehyde (GA) cross-linker	Acetylcholine (Ach)	CV and EIS	-	1.04 µM	[182]
	Rare-earth-doped (lanthanide-doped) upconversion nanoparticles (UCNPs)	Dopamine (DA)	Rayleigh light scattering	0–300 µM	1.62 µM	[183]

#### Femto-biosensors to detect urine-based biomarkers

Urine contains very significant AD biomarkers produced by oxidative degradation of DNA (i.e., 8-hydroxy-2’-deoxyguanosine or 8-OHdG). Zhang et al. have fabricated a highly sensitive fluorescence probe using a specific antibody to detect 8-OHdG with consecutive florescence labeling. The highly sensitive biosensor probe can detect 8-OHdG in urine samples collected from AD transgenic mice with an LOD of 0.18 fM using a capillary electrophoresis laser-induced fluorescence (CE-LIF) detection technique [176].

#### Nano-biosensors to detect urine-based biomarkers

Zengi et al. have reported the mean urinary level of 8-OHdG is 115.7 ± 50 nmol/µmol creatinine in AD patients and 9.28 ± 2.23 nmol/µmol creatinine in the control group. They applied a high performance liquid chromatography-electrochemical detection (HPLC-ECD) technique for the measurement of 8-OHdG biomarker [42]. A group of researchers led by Zhang have evaluated AD7c-NTP, an AD biomarker present in urine, using an ELISA technique. They found that the level of AD7c-NTP was 0.48 ng/mL in a senior age population with lower cognitive function (LCF) and 0.25 ng/mL in normal cognitive controls [17]. Aβ peptide can be detected using polyvinylidene difluoride (PVDF) membrane incubated with primary antibody solution of human Aβ monoclonal antibody [177]. This membrane sensor could detect amyloid biomarker with the help of western blotting technique. Its limit of detection was 0.04 ng/mL. Ghanbari et al. have used a 96-well microtiter plate coated with monoclonal antibody (N3I4) immunoassay to prepare enzyme linked sandwich immunoassay (ELISA). They applied HPLC technique for the measurement of AD7c-NPT biomarker and found that its mean level was 2.5 ng/mL in urine samples of AD patients, higher than that (0.8 ng/mL) in the normal control group [178].

**Fig. 6:**
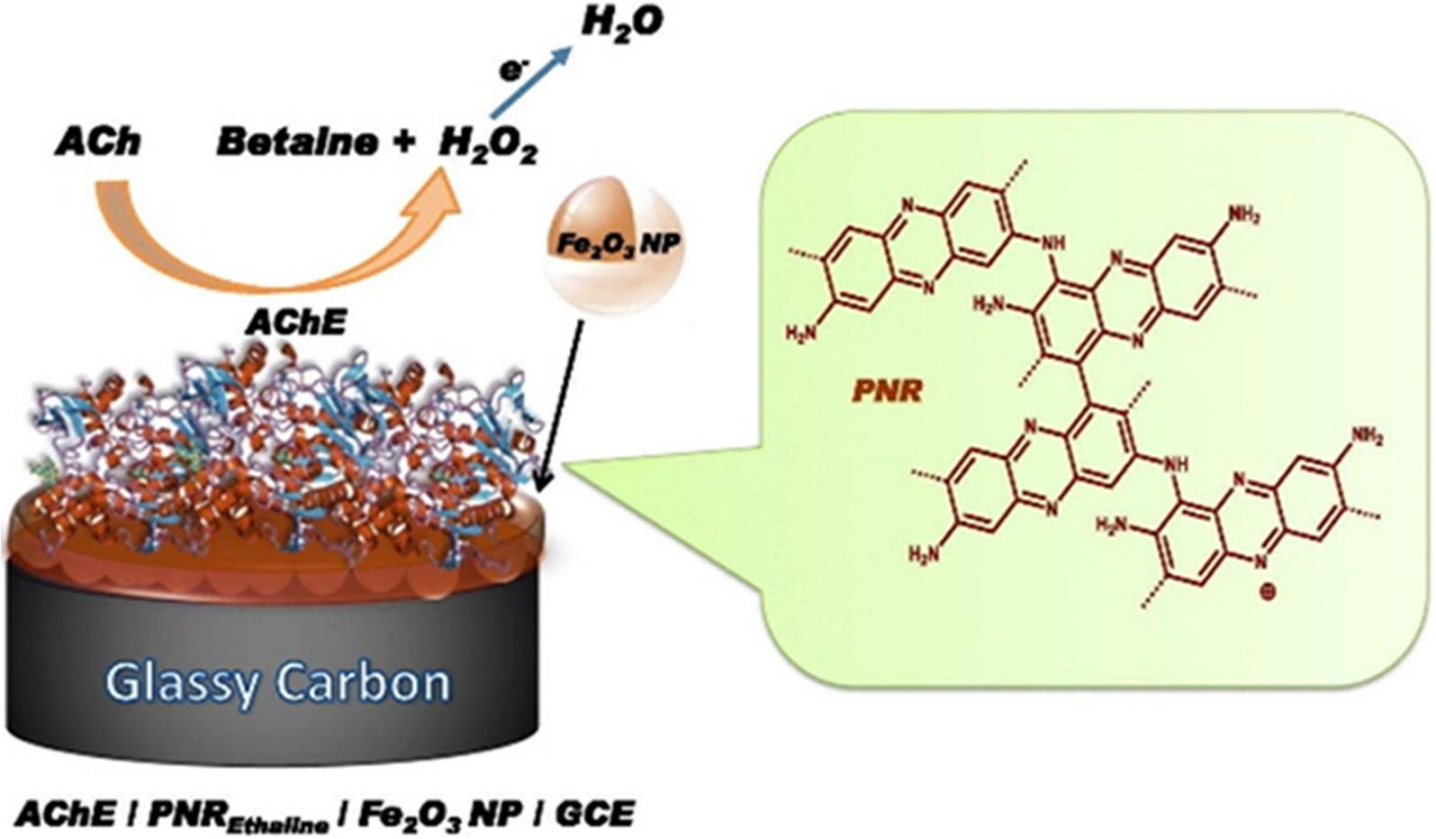
Iron oxide magnetic nanoparticles on poly(neutral red) film for acetylcholine (Ach) biomarker detection from urine. Reprinted with permission from ref. [182]

#### Micro-biosensors to detect urine-based biomarkers

A group of researchers led by Young have measured urine neural thread protein (uNTP) levels using enzyme-linked sandwich immunosorbent assay (ELISA) method. For this process, they utilized a microtiter plate coated with bispecific receptor antibody having affinity for immunoglobulin G (IgG) and urinary NTP as the measurement platform. With such device, the mean uNTP level was measured to be 26.8 ± 9.4 µg/mL in samples collected from AD patients, higher than that (18.1 ± 6.7 µg/mL) in the normal control group [179]. Levy et al. have developed an affinity assay kit using 32-well microtiter plates as enzyme-linked immunosorbent assay for the measurement of neural thread protein (uNTP) using urine samples collected from AD patients. They obtained uNTP levels within a linear range of 10–60 µg/mL with the help of ELISA technique [180]. Ku et al. have determined levels of AD7c-NTP in urine samples collected from AD patients using an enzyme-linked immunosorbent assay technique for the detection of AD7c-NTP. For this purpose, a microtiter plate was coated with a bispecific receptor antibody having high affinities for rabbit immunoglobulin G (IgG) and AD7c-NTP. Using this kit, the mean AD7c-NTP level in MCI patients was measured to be 32.76 ± 9 µg/mL [181]. Figure 6 shows a highly sensitive electrochemical biosensor electrode probe fabricated with a potential cycling electro-polymerization of poly(neutral red) (PNR) film modified with Fe_2_O_3_ magnetic nanoparticles immobilized on acetylcholinesterase (AchE) using glutaraldehyde cross-linking. This biosensor device was able to detect acetylcholine (Ach) using CV and EIS techniques with an LOD of 1.04 µM [182]. Pulgarin et al. have designed a highly sensitive Rayleigh light scattering sensor for the detection of dopamine (DA) from human urine samples. The sensor device was fabricated using rare-earth-doped (lanthanide-doped) upconversion nanoparticles (UCNPs). The sensor showed a linear range of detection of 0–300 µM and an LOD of 1.62 µM with a Rayleigh light scattering method [183].

### Tear-based small biosensors

Similar to urine, tear is also an important biosample with a non-invasive and easy mode of collection for the detection of AD biomarkers [77]. Tears are clear three-layered bio-fluids that cover the frontal part of the eye ball to keep the eye moist and protect the eye ball from foreign particles. Tears are produced by lacrimal glands of the eyelids via blood plasma filtration. Although tear is an excellent source of various electrolytes, proteins, lipids, nucleotides, and biomolecules, studies on the use of tear-based biosensors for the detection of biomarkers of human diseases are limited [184, 185]. Therefore, there is an unmet need for the design and development of ultrasensitive tear-based biosensors for the monitoring and diagnosis of disease conditions of human body using tear samples [186]. Kenny et al. have used tear samples to detect AD-specific protein along with microRNA-based biomarkers. The protein was detected using a liquid chromatography-mass spectrometry (LC-MS) and microRNA was diagnosed with a genome-wide high-throughput polymerase chain reaction-based platform.

**Fig. 7:**
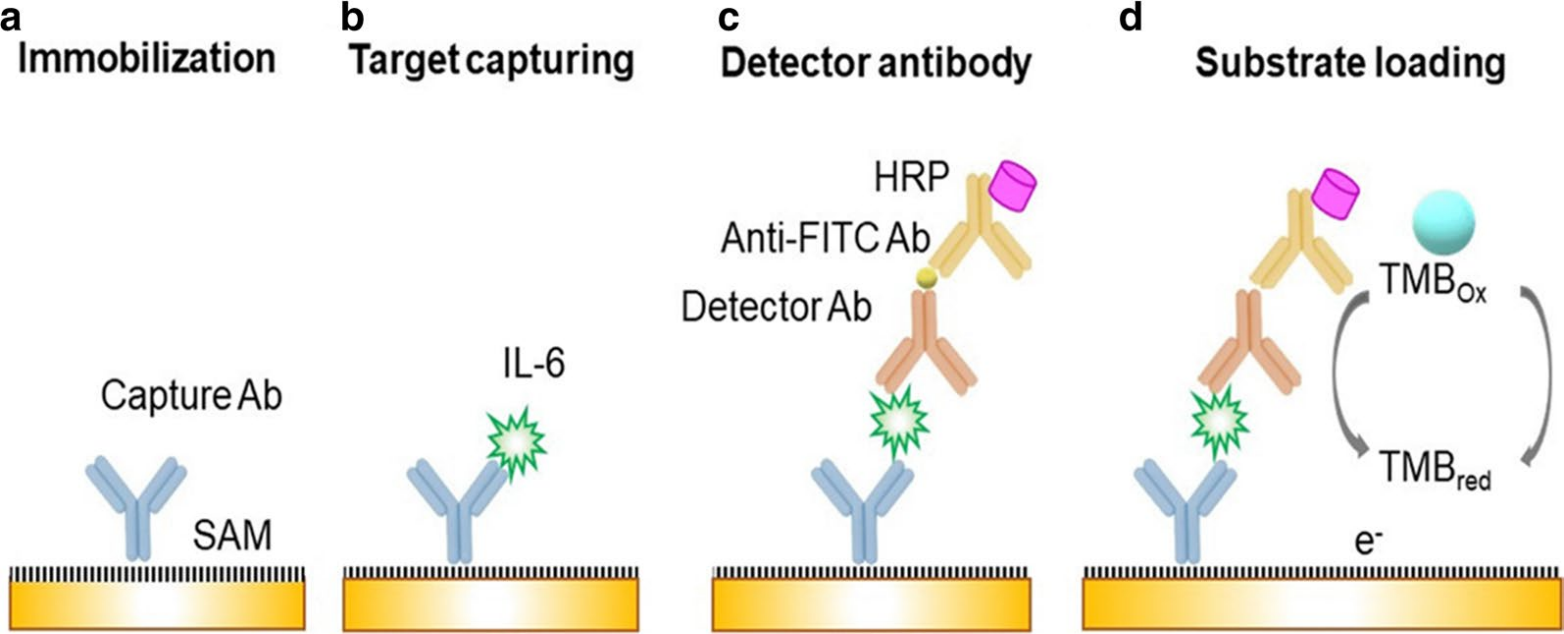
Schematic representations of fundamental steps for biosensor fabrication: **a** Antibody immobilization with a self-assembled monolayer (SAM) on sensor platform, **b** Capture of target cytokine with anti-IL-6 antibody, **c** Functionalization using polyclonal antibodies mixture and conjugated anti-fluorescein isothiocynate (anti-FITC) antibody loading with horseradish peroxide, and **d** Amperometric signal from enzymatic amplification cycle of substrate. Reprinted with permission from ref. [187]

#### Femto-biosensors to detect tear-based biomarkers

Until now, there are no documented results in the web library for the detection of AD biomarkers from tear-based samples using femto-level biosensors. Thus, biosensor researchers have plenty of opportunity to focus on the development of highly useful femto-scale biosensors to enhance early diagnosis of AD.

#### Nano-biosensors to detect tear-based biomarkers

Some researchers have worked on nano-level detection of AD biomarkers from tear samples. Punj et al. have successfully detected levels of multifunctional chemical messengers known as cytokines with an efficient electrochemical biosensor platform using the amperometric method for measuring chemical conductance as shown in Fig. 7. Immune-analysis of the tear fluids showed a limit of detection of 0.0221 ng/mL [187].

#### Micro-biosensors to detect tear-based biomarkers

Kallo et al. have detected tear-based proteins from tear samples collected from AD patients and healthy controls. They reported an increase in the concentration of tear protein (8.8 ± 2.9 µg/µL) in AD patients compared to that in normal controls (4.4 ± 1.4 µg/µL). For the measurement of tear proteins, they used the standard method of quantitative proteomics followed by electrophoresis and liquid chromatography-mass spectroscopy/mass spectroscopy (LC-MS/MS) [188].

### Sweat-based small biosensors

Sweat is a body fluid produced by the skin. It is an electrolyte-rich salty solution [189]. Sweat is generally used for electrochemical detection of different metabolites such as uric acid and glucose. It is rarely used for the detection of proteomics because it contains very low concentrations of sweat proteins. This is due to the lower permeability of large protein molecules [190]. Technological advancement has led to the development of stretchable, biocompatible, and wearable electronic tattoo-like devices for sweat analysis [191].

#### Femto-biosensors to detect sweat-based biomarkers

Of all biosamples, sweat is seldom used as a sample for the diagnosis of AD diseases because it is a very dilute aqueous solution with very low concentrations of proteomics and biomolecules associated with AD. Therefore, a highly sophisticated, advanced, and ultrasensitive femto-level biosensor is required to use sweat for the diagnosis of AD.

#### Nano-biosensors to detect sweat-based biomarkers

**Fig. 8:**
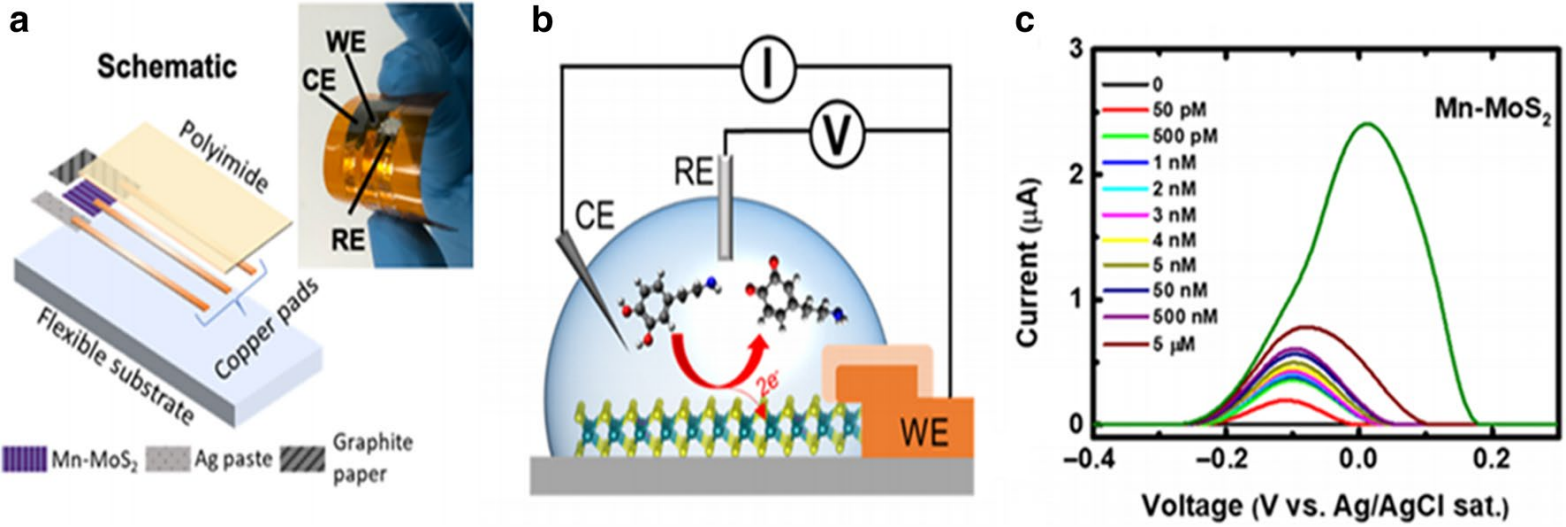
Fabrication of sweat-based biosensor and its working principle: **a** An integrated sensor with working (WE), reference (RE), and counter electrode (CE) for DA detection, **b** A graphical presentation of testing setup, and (C) DPV graph obtained from the DA sensor. Reprinted with permission from ref. [192]

Until now, there have been few technological advancements showing the applicability of nano-level biosensor for the detection of AD biomarkers from sweat samples. AD patients are known to have lower concentrations of dopamine (DA) in sweat. Lei et al. have formulated a single-atom doped MoS_2_ with Mn for the ultrasensitive detection of DA from an artificial sweat sample, showing a limit of detection of 0.05 nM with the application of cyclic voltammetry and EIS techniques. This may provide a new strategy for pathological diagnosis of AD with sweat as shown in Fig. 8 [192].

#### Micro-biosensors to detect sweat-based biomarkers

Sodium ion present in sweat sample could be used to monitor the health status of AD patients. Elmstahl et al. have estimated that total sodium concentration in sweat samples collected from AD patients using iontophoresis method is 91 µm/mL, higher than that in normal controls [193].

### Saliva-based small biosensors

Saliva is the most attractive non-invasive oral matrix could be used as an ideal source of biomarkers for the detection of AD. The use of saliva as a biosample for early detection of AD has grown with exponential rate in the last decades. The literature shows that it contains majority of proteins found in blood stream and used as an excellent source for the omics profiling process for the qualitative and quantitative characterization of saliva proteins also known as “salvaomics” [194]. There are different techniques like ELISA, qRT-PCR, Luminex assay, antibody conjugated magnetic nanoparticles, mass spectrometry etc. used for the detection of Aβ present in saliva [195]. Aβ42 levels were recorded to almost double in the AD and MCI patients compared to normal controls. The value of Aβ42 when exceeds the level 40 pg/mL in the salivary secretion than it is regarded to induces the early pathogenic conditions of AD or MIC conditions [196]. Similarly, tau concentration present in salivary is also of little significance, but the higher value of ratio of p-tau/tau is observed in AD and MCI patients [9]. Stem cells present in saliva are aged prematurely and disrupted with the age-factors which could be used for the detection of cell abnormality and correlated for the beta-amyloid plaque formation, a main cause of neurodegenerative disorder appears as AD [66, 197]. The salivary epithelial cells collected from AD patients demonstrated the alternation of amyloid precursor proteins (APPs) which is supposed to contribute the accumulation of β-amyloid peptides [198]. Sabbagh et al. used enzyme-linked immunosorbent assay for the quantitative analysis of Aβ42 in saliva sample collected from AD patients and recorded as 51.7 ± 1.6 pg/mL which is higher than that of the control group [199]. The conflicting result was reported with saliva as biosamples. Various methods for the analysis of AD biomarkers in saliva-based biosamples still do not show consistent results. The proper quantification and presentation of different biomarkers that are present in very low concentration in saliva must be standardized to eliminate the errors during the accurate identification of saliva-based biomarkers. Therefore, further intensive research and investigation should be conducted for the identifications and validations of protocols with the available diagnostic tools for the early detection and proper management of AD using saliva as non-invasion biosample.

### Future trends in the development of biosensors

Since there is no therapeutic drug that can stop the progression of AD or completely recover AD, early diagnosis of AD is very important. After early diagnosis, symptoms of AD can be alleviated and the progression of AD can be slowed through appropriate treatment. Due to this reason, femto-, nano-, and micro-scale biosensing technologies using biosamples are highly promising tools for early diagnosis of onset and progression of AD.

The most significant disadvantage of CSF and blood-based detection is associated with the invasive method of sample collection. To overcome the current limitation of invasive sampling, the new trend in research is focused on the development of new non-invasive techniques such as an optical-screening or a bio-imaging process for AD diagnosis. Baksh et al. have used coherent extreme UV (EUV) from an ultrafast laser to create images of samples from mouse hippocampal neurons with the potential for high-resolution element-specific 3D imaging within biological structures. This non-invasive technique has potential applications in AD diagnosis. Kawasaki et al. have also reported a far-infrared (FIR) free-electron laser (FEL) for detecting amyloid protein aggregates in brains of AD patients [200].

Although non-invasive biosamples such as urine, tears, and sweat have been used for the detection of AD biomarkers, low concentrations of AD biomarkers in these samples have limited their use for early detection of AD. For this reason, researchers have made efforts to discover and detect new types of biomarkers from non-invasive body fluids to enhance sensitivities of biosensors. Yan et al. have leveraged a modern technique known as small-input liquid volume extracellular RNA sequencing (SILVER-seq) to generate extracellular RNA (exRNA) profiles of human plasma samples. They discovered that phosphoglycerate dehydrogenase (PHGDH), a unique gene, had consistently higher levels in AD brain transcriptomes and plasma samples from three independent cohorts [201]. Their finding suggests that overproduction of exRNA by PHGDH gene in elderly people could provide an early warning for the development of AD. Similarly, Berdyyeva et al. have discovered a novel AD biomarker for PET imaging tracer ‘(^18^F)JNJ-64413739 in a rat model [202]. It can act as a potential PET tracer of central neuro-inflammation to aid the control and management of AD before its pathological onset.

Future biosensing techniques for AD diagnosis can be combined with wearable devices (skin patch, tattoo, masks, contact lenses, wrist watches, and so on) that may also be connected with remote systems for continuous monitoring of patients’ health status. Furthermore, when combined with artificial intelligence (AI), the accuracy of AD diagnosis can be further improved. Rapid technological advancement had led to the emergence of digital biomarkers and digital biosensors for early detection and diagnosis of Alzheimer’s disease. A digital biomarker refers to objective and strategic physiological behavioral data collected and measured through wearable and implantable digital devices [203, 204]. Digital biosensors represent bioanalysis systems logically processed with multiple biochemical signals [205]. The future is facing an inflection point in medical diagnosis of AD with simultaneous emergence of digital biomarkers and biosensors. Multisignal-based digital biomarkers and biosensors can have a profound impact on early diagnosis of AD.

## Conclusions

We reviewed current development, challenges, and future directions of femto-, nana-, and micro-biosensors using invasive and non-invasive biosamples for early detection of AD. Based on the detection scale from femto to micro levels, analytical performances of small biosensors were summarized depending on specific body fluids (CSF, blood, urine, tear, and sweat) containing AD biomarkers. Also, we summarized detailed sensing techniques to ultrasensitively detect multiple target biomarkers (i.e., Aβ peptide, tau protein, Ach, etc.) of AD with regards to detection ranges and limits of detection. We extensively introduced future trends of new noninvasive detection techniques (such as optical screening and bio-imaging process) to hurdle current issue associated with the invasiveness of sample collection (CSF and blood). In addition, discovery of new types of AD biomarkers from non-invasive body fluids will be challenging for early diagnosis of AD. The future will face an inflection point in early diagnosis of AD with simultaneous emergence of addressable innovative technologies such as new non-invasive detection methods, wearable devices, and new types of ultrasensitive biomarkers, digital biomarkers, and digital biosensors.
