# Correction to: Advances in the development paradigm of biosample‐based biosensors for early ultrasensitive detection of alzheimer’s disease

**DOI:** 10.1186/s12951-021-00850-3

**Published:** 2021-04-26

**Authors:** Hem Prakash Karki, Yeongseok Jang, Jinmu Jung, Jonghyun Oh

**Affiliations:** 1 Department of Mechanical Design Engineering, College of Engineering, Jeonbuk National University, Jeonju, 54896 South Korea; 2 Department of Nano-bio Mechanical System Engineering, College of Engineering, Jeonbuk National University, Jeonju, 54896 South Korea

## Correction to: J Nanobiotechnol (2021) 19:72 https://doi.org/10.1186/s12951-021-00814-7

Following publication of the original article [1], the authors identified inadvertent errors in caption of Fig. 4.

The caption for Fig. 4 originally read:

Ultrasensitive detection of amyloid-beta-oligomers in mice CSF using a poly(3,4-ethylene dioxythiophene) (PEDOT)-embedded Au nanoelectrode. Reprinted with permission from ref. [138].

The caption for Fig. 4 should read: Figure 4 Conceptual illustration for the detection of Aβ42 using localized surface plasmon resonance (LSPR) technique. Reprinted with permission from Ref. [138]

The original article [1] has been corrected.
